# Dataset on the beneficiation of a Nigerian bentonite clay mineral for drilling mud formulation

**DOI:** 10.1016/j.dib.2018.07.071

**Published:** 2018-08-03

**Authors:** Richard O. Afolabi, Temitope F. Ogunkunle, Oluwasanmi A. Olabode, Esther O. Yusuf

**Affiliations:** a Department of Petroleum Engineering, Covenant University, P.M.B 1023 Ota, Ogun State, Nigeria; b Department of Chemical Engineering, Covenant University, P.M.B 1023 Ota, Ogun State, Nigeria

## Abstract

This paper presents dataset on the beneficiation of a Nigerian clay mineral for drilling mud application. The experimental design applied used a Response Surface Design (RSM), which involved 2^4^ (2-Level, 4-Factors) to generate statistical models, and analyze the dataset. The independent variables were (Bentonite; *X*_1_), (Polymer; *X*_2_), (Sodium Carbonate, *X*_3_) and (Aging Time; *X*_4_). The rheological properties of interest, which forms the response variables, were selected based on the API specification 13-A for drilling grade bentonite. The outcomes show that the second-order statistical models derived from responses fitted well with the experimental results. Predictive models obtained from the statistical characterization of the beneficiation process would allow for the design and cost-effective planning of the procedure. The beneficiation of the clay using sodium carbonate and Kelzan^®^ XCD polymer ensued in an improvement in the rheological properties of the formulated drilling mud. These properties were comparable with the API specification 13-A for drilling fluid materials.

## Specifications Table

**Table:** 

Subject area	*Petroleum Engineering*
More specific subject area	*Bentonite Clay Chemistry/Drilling Fluid Technology*
Type of data	*Tables and Figures*
How data was acquired	*Rheological Study using the OFITE*^*®*^*Model 800 Viscometer*
Data format	*Raw Data*
Experimental factors	1. *Beneficiation of a locally sourced Nigerian bentonite clay using sodium carbonate and Kelzan XCD polymer*. 2. *A rheological analysis carried out using OFITE®* Model 800 Viscometer.
Experimental features	1. *Improvement in the rheological properties of drilling mud made from the beneficiated clay when compared to the raw clay*. 2. *The properties obtained from the prepared drilling mud were comparable to the API specification 13-A*.
Data source location	*Department of Petroleum Engineering, Covenant University, Nigeria*
Data accessibility	*Data is with the article*
Related research article	*None*

## Value of the data

• Dataset shows the relevance and significance of the beneficiation of Nigerian bentonite clays for drilling application in the petroleum industry. Hence, an all-inclusive approach is required towards the characterization, rheological study and beneficiation of the various clay deposits in Nigeria [1], [2], [3]. This would provide a comprehensive overview of the vast deposits of clay under consideration and approach necessary for its exploitation [3].
• The rheological properties of drilling mud obtained from the beneficiated clays where comparable with the API specification 13-A for drilling grade bentonite. This is significant as the present level of consumption of bentonite for drilling operations is high, most of which are imported in the Nigerian oil and gas industry [2], [3], [4], [5].
• Predictive models obtained from the statistical characterization of the beneficiation process would allow for the design and cost-effective planning of the procedure. This would allow for detailed comparison between the costs of the beneficiation procedure as against the cost of importing drilling grade bentonite [2], [3], [6].
• The dataset indicates that optimal determination of the quantitative amounts of the beneficiating additives required to standardize Nigerian bentonite clays to the API drilling grade specification is necessary [3].

## 1 Data

**Fig. 1:**
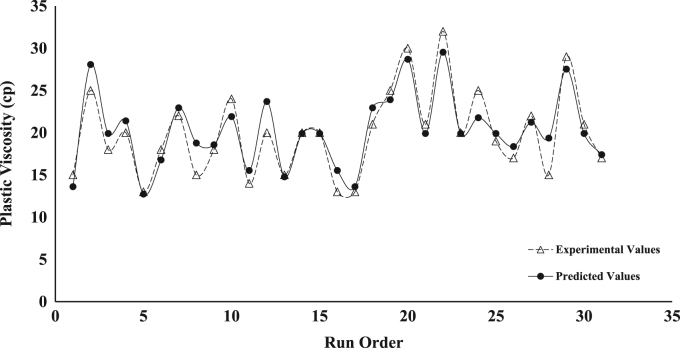
Comparison between the values obtained for the predictive statistical model for plastic viscosity and the experimentally obtained values.

**Fig. 2:**
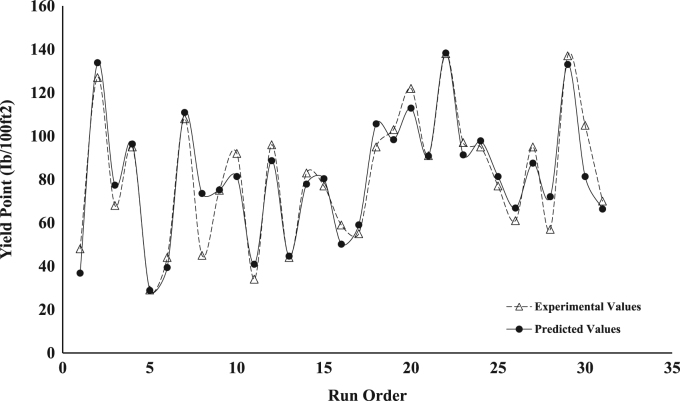
Comparison between the values obtained for the predictive statistical model for yield point and the experimentally obtained values.

**Fig. 3:**
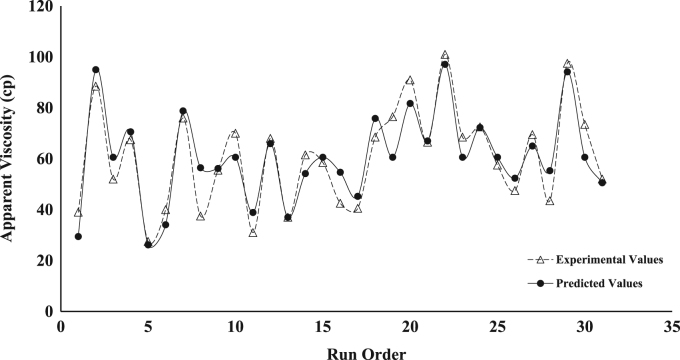
Comparison between the values obtained for the predictive statistical model for apparent viscosity and the experimentally obtained values.

**Fig. 4:**
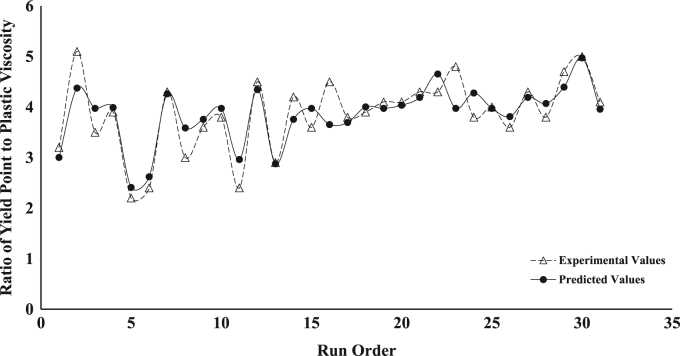
Comparison between the values obtained for the predictive statistical model for the ratio of yield point to plastic viscosity and the experimentally obtained values.

**Fig. 5:**
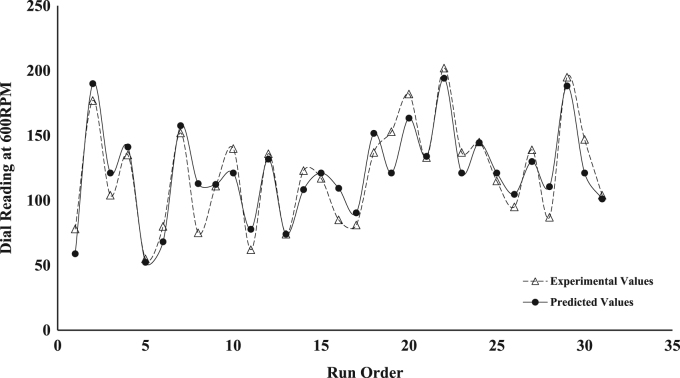
Comparison between the values obtained for the predictive statistical model for viscometric dial reading at 600-RPM and the experimentally obtained values.

The dataset presented represents the statistical characterization of the beneficiation procedure employed in improving the rheological properties of drilling mud prepared from a Nigerian bentonite clay. Tables 1 and 2 represent the factor level settings employed for the various predictors and the 2^4^ (2-Level, 4-Factors) Central Composite Design (CCD) experimental matrix respectively. Table 3 contains a summary of the properties for drilling grade bentonite as contained in the API specification 13-A [6]. Table 4 shows the Analysis of Variance (ANOVA) used in testing for the significance of the predictors (or independent variables) on the beneficiation process (ratio of Yield Point to Plastic Viscosity, *YP*/*PV*, was taken as response variable of interest). Table 5 contains values for the parameter constants describing the predictive statistical models obtained from the regression analysis. Fig. 1, Fig. 2, Fig. 3, Fig. 4, Fig. 5 show a comparison between the values obtained for the predictive statistical models describing the rheological properties and the experimentally obtained values.

**Table 1:** Independent variables and their levels used for Central Composite Design (CCD) design.

**Independent variable**	**Level**				
	**− 2**	**− 1**	**0**	**1**	**2**
Bentonite Content, g (*X*_1_)	20	25	30	35	40
Polymer Content, g (*X*_2_)	2.5	5	7.5	10	12.5
Sodium Carbonate, g (*X*_3_)	2	4	6	8	10
Aging Time, h (*X*_4_)	5	10	15	20	25

**Table 2:** Experimental design table for the beneficiation of the obtained Ewekoro clay using Kelzan^®^ XCD polymer and sodium carbonate.

		**Independent variables**				**Response variables**					
**Standard order**	**Run order**	***X***_**1**_	***X***_**2**_	***X***_**3**_	***X***_**4**_	***PV***	***AV***	***YP***	η	***YP*/*PV***	$\theta_{600}$
11	1	−1	1	**−** 1	1	15	39.0	48	2450	3.2	78
18	2	2	0	0	0	25	88.5	127	7900	5.1	177
25	3	0	0	0	0	18	52.0	68	2600	3.5	104
22	4	0	0	2	0	20	67.5	95	4200	3.9	135
17	5	−2	0	0	0	13	27.5	29	1200	2.2	55
5	6	−1	**−** 1	1	− 1	18	40.0	44	2200	2.4	80
8	7	1	1	1	− 1	22	76.0	108	2400	4.3	152
12	8	1	1	**−** 1	1	15	37.5	45	2000	3.0	75
23	9	0	0	0	− 2	18	55.5	75	2700	3.6	111
30	10	0	0	0	0	24	70.0	92	3000	3.8	140
3	11	−1	1	−1	−1	14	31.0	34	1600	2.4	62
4	12	1	1	−1	−1	20	68.0	96	5100	4.5	136
7	13	− 1	1	1	**−** 1	15	37.0	44	2300	2.9	74
20	14	0	2	0	0	20	61.5	83	4200	4.2	123
28	15	0	0	0	0	20	58.5	77	4000	3.6	117
1	16	−1	**−** 1	**−** 1	**−** 1	13	42.5	59	2400	4.5	85
9	17	**−** 1	−1	−1	1	13	40.5	55	2100	3.8	81
6	18	1	−1	1	**−** 1	21	68.5	95	4800	3.9	137
26	19	0	0	0	0	25	76.5	103	5600	4.1	153
2	20	1	**−** 1	−1	**−** 1	30	91.0	122	6600	4.1	182
19	21	0	−2	0	0	21	66.5	91	4600	4.3	133
16	22	1	1	1	1	32	101.0	138	7400	4.3	202
29	23	0	0	0	0	20	68.5	97	4800	4.8	137
10	24	1	−1	**−** 1	1	25	72.5	95	5200	3.8	145
27	25	0	0	0	0	19	57.5	77	3600	4.0	115
13	26	−1	**−** 1	1	1	17	47.5	61	3900	3.6	95
24	27	0	0	0	2	22	69.5	95	6900	4.3	139
15	28	**−** 1	1	1	1	15	43.5	57	2200	3.8	87
14	29	1	−1	1	1	29	97.5	137	7200	4.7	195
31	30	0	0	0	0	21	73.5	105	5600	5.0	147
21	31	0	0	− 2	0	17	52.0	70	3300	4.1	104

**Table 3:** Drilling grade bentonite clay as specified in American Petroleum Institute (API) specification 13-A.

**Requirements**	**Specification**
Reading at 600 RPM^a^	30, minimum
Reading at 300 RPM^a^	24, maximum
*PV*^b^	–
*YP*^c^	–
*YP*/*PV* ratio^a^	3, maximum

a API Requirements.

b $\mathrm{PV}=\theta_{600}-\theta_{300}$.

c $\mathrm{YP}=\theta_{300}-\mathrm{PV}$.

**Table 4:** ANOVA table for the fitted second-order statistical model describing *YP*/*PV*^a^. The significance of the various variables on the beneficiation process is captured using YP/PV as the response variable.

**Source**		**D*F***	**Adj. SS**	**Adj. MS**	***F*-value**	***P*-value**	**Significance**
**Model**		5	9.4542	1.89084	44.77	0.000	**
	**Linear**	4	9.2300	2.30750	54.64	0.000	**
	***X***_**1**_	1	0.9000	0.90000	21.31	0.000	**
	***X***_**2**_	1	7.2250	7.22500	171.1	0.000	**
	***X***_**3**_	1	0.5290	0.52900	12.53	0.003	**
	***X***_**4**_	1	0.5760	0.57600	13.64	0.002	**
**Square**		1	0.2242	0.22422	5.31	0.037	*
	***X***^**2**^	1	0.2242	0.22422	5.31	0.037	*
**Error**		14	0.5913	0.04223	–	–	
	**Lack of Fit**	11	0.5913	0.05375	–	–	
	**Pure Error**	3	0.0000	0.00000	–	–	
**Total**		19	10.045	–	–	–	

a $\mathrm{YP}/\mathrm{PV}=1.55-0.1510X_{1}+0.3400X_{2}+0.1150X_{3}+0.0480X_{4}+0.00352X_{1}^{2}$ ($R^{2}$ = 0.9411, $R_{\mathrm{adj}}^{2}$ = 0.9211, $R_{\mathrm{pred}}^{2}$ = 0.7996).

* Significant (*P* < 0.05).

** Extremely Significant (*P*≤ 0.03).

**Table 5:** Parameter values for the statistical model describing the various rheological properties of the drilling mud prepared from the beneficiated bentonite clay.

***Y***	$\beta_{o}$	$\beta_{1}$	$\beta_{2}$	$\beta_{3}$	$\beta_{4}$	$\beta_{11}$	$\beta_{22}$	$\beta_{33}$	$\beta_{44}$	$\beta_{12}$	$\beta_{13}$	$\beta_{14}$
*PV*	19.90	1.25	4.08	0.67	–	–	–	–	–	–	1.63	–
*YP*	81.35	7.50	26.25	4.75	3.08	–	–	–	–	6.87	7.37	10.63
*AV*	60.58	5.00	17.21	3.21	2.21	–	–	–	–	3.69	4.69	6.94
*YP*/*PV*	1.55	0.15	0.34	0.12	0.05	0.0035	–	–	–	–	–	–
$\theta_{600}$	121.16	10.00	34.42	6.42	4.42	–	–	–	–	7.37	9.37	13.88

## 2 Experimental design, materials, and methods

### 2.1 Materials

The local bentonite clay used for this study was a raw, non-treated bentonite clay sourced from sourced from *Ewekoro* (6°56′N 3°13′E) in Ogun State, South-West Nigeria. Clay storage condition in the laboratory was at room temperature.

The polymer employed is a dispersible, high molecular weight xanthan gum biopolymer with trade name Kelzan^®^ XCD Polymer. This was purchased from Equilab solutions, Nigeria. The physical properties of the polymer include Appearance: white-tan powder, Physical state: solid and odorless, Specific gravity of 1.5 at a temperature of 25 °C, good solubility in water and a pH of 7.0 (1 wt% solution).

Sodium carbonate, ${\mathrm{Na}}_{2}{\mathrm{CO}}_{3}$, (product number: 791768) manufactured by Sigma Aldrich and with the following properties was used: white appearance and powder form, calcium content (<0.03%), iron content (<5 ppm), lead content (<5 ppm), potassium content (<0.005%), magnesium content (< 0.005%), chloride (< 0.001%), phosphate content (<0.001%), sulphur content (<0.003%), silica content (<0.005%). The sodium carbonate was also purchased from Equilab solutions.

### 2.2 Beneficiation procedure of local bentonite clay

The beneficiation procedure followed was similar to what was adopted by [7]. The clay sample lumps were crushed and large particles such as pebbles and dirt were removed. The crushed clay was screened with 2 mm sieves to remove stones and other coarse particles. The resulting material was finally dried in an oven at 150–200 °C to remove moisture, volatile and readily combustible organics in the sample. The clay sample was then milled and screened with 150 μm sieve. Sodium saturation of the clay involved placing 25–50 g of screened sample (with 2 mm sieve) in 100 cm^3^ of a solution containing 2–10 g Na_2_CO_3_. After stirring for 30 min, the pH of the suspension was adjusted to 11–12 with 0.1 M NaOH. The suspension was further stirred for another 30 min and allowed to age for 5–48 h. Clay particles (size, 2 mm) in the suspension were then extracted by repeated sedimentation and siphoning. The resulting samples obtained were air dried and kept at room condition. The air-dried samples were treated with 5–12.5 g of Kelzan^®^ XCD Polymer to improve the rheology of the beneficiated bentonite clay. These operating conditions were selected after single factor experiment as shown Table 1.

### 2.3 Statistical design of beneficiation process

The procedure was carried out using a 2^4^ (2-Level, 4-Factors) CCD to generate a statistical model for the analysis of quadratic effects and interaction effects between the independent variables: bentonite clay, polymer, sodium carbonate and the aging time. The 2^4^ CCD contains 31 experimental runs as contained in Table 2. The four variables chosen for the 2^4^ CCD were designated as (Bentonite; *X*_1_), (Polymer; *X*_2_), (Sodium Carbonate, *X*_3_) and (Aging Time; *X*_4_). The statistical tool, MINITAB^®^ 17 (PA, USA), was used for the experimental design, and statistical analyses of the laboratory data. The rheological properties, which form response variables, were fitted to the polynomial model in Eq. (1) using regression analysis.

(1) $$Y=\beta_{o}+\sum_{i=1}^{4}\beta_{i}X_{i}+\sum_{i=1}^{4}\beta_{\mathrm{ii}}{X_{i}}^{2}+\sum_{i=0}^{3}\sum_{j=i+1}^{4}\beta_{\mathrm{ij}}X_{i}X_{j}$$

Y: the predicted response; $\beta_{o}$: the intercept coefficient; $\beta_{i}$: the linear coefficient; $\beta_{\mathrm{ii}}$: the squared coefficient; $\beta_{\mathrm{ij}}$: the interaction coefficient; $X_{i}$ $X_{j}$: the coded independent variables; $X_{i}X_{j}$: the interaction terms; ${X_{i}}^{2}$: the quadratic terms.

### 2.4 Response variable for the beneficiation process

The physical requirements of a drilling grade bentonite clay as specified in American Petroleum Institute (API) specification 13-A is shown in Table 3. The ratio of Yield Point to Plastic Viscosity (*YP*/*PV*) was taken as the response variable. The estimation of this ratio also requires the other API parameters listed in Table 3. The PV and YP were calculated using Eqs. (2), (3), (4) below:

(2) $$\mathrm{Plastic}\;\mathrm{Viscosity}\;\left(\mathrm{PV}\right)\;=\;\theta_{600}\;-\;\theta_{300}$$

(3) $$\mathrm{Yield}\;\mathrm{Point}\;\left(\mathrm{YP}\right)\;=\;\theta_{300}\;-\;\mathrm{PV}$$

(4) $$\mathrm{Apparent}\;\mathrm{Viscosity}\;\left(\mathrm{AV}\right),\;\mathrm{cp}\;=\;\theta_{600}/2$$

$\theta_{600}$ and $\theta_{300}$ represents the viscometer dial readings at 600-RPM and 300-RPM respectively. The OFITE Model 800 (8-Speed) Viscometer was used to obtain dial readings, θ, at various RPM values (3, 6, 30, 60, 100, 200, 300, and 600). The ANOVA table for the *YP*/*PV* is shown in Table 4. Viscosity data were estimated by applying (5) as indicated in the OFITE working guide (1 cp is equal to $10^{-3}\;\mathrm{Pa}\;s$).

(5) $$\eta =\mathrm{KF}\frac{\theta }{\mathrm{RPM}}$$

η is the viscosity in cp, spring factor, *F* = 1 (R1B1 combination), machine constant, *K* = 300 (R1B1 combination).
